# Considerations and Regulatory Approaches in the USA and in the EU for dsRNA-Based Externally Applied Pesticides for Plant Protection

**DOI:** 10.3389/fpls.2021.682387

**Published:** 2021-06-11

**Authors:** Antje Dietz-Pfeilstetter, Mike Mendelsohn, Achim Gathmann, Dominik Klinkenbuß

**Affiliations:** ^1^Federal Research Centre for Cultivated Plants, Institute for Biosafety in Plant Biotechnology, Julius Kühn-Institut, Braunschweig, Germany; ^2^Biopesticides and Pollution Prevention Division, Office of Pesticide Programs, United States Environmental Protection Agency, Washington, DC, United States; ^3^Federal Office of Consumer Protection and Food Safety, Department of Plant Protection Products, Unit Environment, Braunschweig, Germany

**Keywords:** plant protection, pesticide, dsRNA, SIGS, pesticide regulation

## Abstract

Increasing pest and pathogen challenges as well as having fewer conventional pesticides to employ require innovative and sustainable solutions for plant protection. One group of pesticides that is in the pipeline and is expected to be subject to regulation and risk assessment procedures in the near future, is based on the natural gene silencing mechanism RNA interference (RNAi). These dsRNA-based products can be highly specific for a target organism due to the sequence-specific interaction between effective small interfering RNAs (siRNAs) and a complementary target RNA. General regulatory frameworks for pesticide authorization in the U.S. and in the EU are presented. In addition, production and application procedures and specific characteristics of dsRNA-based pesticides relevant for risk assessment and regulation are considered.

## Introduction

Increasing food demands of a growing world population in combination with new plant pests and diseases due to climate change (Chakraborty and Newton, 2011) call for improved solutions for crop protection. The future potential of current pest and pathogen control in crops based on chemical pesticides is limited because fewer approved pesticides are available, especially in Europe. Actually, it is expected that the “Green Deal” recently initiated in the European Union (EU) will result in a further reduction of active pesticidal substances for the control of insect pests and fungal pathogens in Europe. Therefore, innovative and sustainable approaches are required to combat current and future pest problems.

One possible solution is the cultivation of genetically modified (GM) crops with built-in plant protection. Examples are GM crops expressing toxins from the soil bacterium *Bacillus thuringiensis* (Bt) that specifically kill certain pest insects (e.g., Perlak et al., 1990) and GM maize expressing double-stranded (ds) RNA resulting in the sequence specific degradation of messenger RNA (mRNA) coding for an essential insect protein by an RNA interference (RNAi) mechanism (Bachman et al., 2016). GM crops with such plant-incorporated protectants (PIP) have been authorized for cultivation in the USA several years ago (Mendelsohn et al., 2003; Head et al., 2017). In the EU, however, there is only cultivation of one insect resistant Bt maize line on small hectarages in Spain and Portugal (Gómez-Barbero et al., 2008; ISAAA, 2019).

RNAi, which is a natural gene silencing mechanism in plants and animals (Fire et al., 1998; Baulcombe, 2004) can not only be used for plant-incorporated protection in GM crops by means of host-induced gene silencing (HIGS), but can also serve as a new mode of action for exogenously applied pesticides. There have been various reports of RNAi molecules as plant protection compounds (San Miguel and Scott, 2016; Worrall et al., 2019) since the first successful foliar applications of dsRNAs for plant virus control (Tenllado and Diaz-Ruiz, 2001). Fungal pathogens as well as different insect pests are susceptible to RNAi and can be controlled by topical dsRNA applications (Fletcher et al., 2020; Taning et al., 2020). RNAi is a fast-growing technology with increasing commercial interest, which is reflected by global research trends and the patent situation (Jalaluddin et al., 2019). It is expected that RNAi-based plant protection products, which are much more specific than conventional pesticides and less persistent in the environment, will be available in the near future for practical uses and will need to undergo appropriate safety evaluation and authorization procedures.

## United States (U.S.) Regulatory Framework for Pesticides

In the U.S., a pesticide is defined as: (1) any substance or mixture of substances intended for preventing, destroying, repelling, or mitigating any pest; (2) any substance or mixture of substances intended for use as a plant regulator, defoliant, or desiccant; and (3) any nitrogen stabilizer. Pesticide products must obtain a U.S. Environmental Protection Agency (EPA) registration before manufacture, transport, and sale (Leahy et al., 2014). Field testing prior to a registration requires an Experimental Use Permit. EPA assesses pesticide end-use and manufacturing-use products for registrations and evaluates the active ingredient as well as the actual products. Additionally, states or territories within the U.S. require pesticide product registration as well. While most states or territories rely on the review of data and safety findings of the U.S. federal government, there are several states, e.g., California, that also require data to be submitted to them, which they review and also do their own assessment.

EPA primarily regulates the use of pesticides under the legal basis of two federal statutes enacted by the U.S. Congress: the Federal Insecticide, Fungicide, and Rodenticide Act (FIFRA) and the Federal Food, Drug, and Cosmetic Act (FFDCA). Other statutes that play roles in the regulation of pesticides in the U.S. include the Food Quality Protection Act of 1996 (FQPA), the Pesticide Registration Improvement Act (PRIA), the Endangered Species Act (ESA), the Migratory Bird Treaty Act, and the Clean Water Act. FIFRA is the pesticide licensing law and provides the basis for registration, sale, distribution, and use of pesticides in the U.S. FIFRA authorizes EPA to assess and register pesticides for specific uses. EPA also has the authority to suspend or cancel the registration of a pesticide if subsequent information shows that continued use would pose unreasonable risks. Pesticide registration is based on a risk/benefit standard. Through the registration process, EPA regulates pesticide use through labeling, packaging, composition, and disposal.

EPA must make a finding of no unreasonable adverse effects to man and the environment from use of the pesticide in order to support its registration decision under FIFRA. The Federal Food, Drug, and Cosmetic Act (FFDCA) authorizes EPA to set maximum residue levels, or tolerances, for pesticides used in or on foods or animal feed. Under FFDCA and amendments to both FFDCA and FIFRA under the FQPA, EPA must make a similar finding of a reasonable certainty of no harm if the use of such agents results in residues in food or feed. If the submitted information supports this safety finding, EPA may establish a numerical tolerance or an exemption from the requirement of a tolerance regarding those residues.

Data requirements for assessing pesticide products are laid down in the regulations enacted by EPA in Title 40 of the Code of Federal Regulations (CFR) Part 158. These data requirements support evaluation of both the pesticide active ingredients and products. In addition to data requirements for conventional chemical pesticides, 40 CFR Part 158 lists requirements for biochemical and microbial pesticides which are usually inherently less toxic than conventional chemical pesticides. Biochemical pesticides are naturally occurring substances that control pests by non-toxic mechanisms. Conventional pesticides, by contrast, are generally synthetic materials that directly kill or inactivate the pest. Biochemical pesticides include substances that interfere with mating, such as insect sex pheromones, as well as various scented plant extracts that attract insect pests to traps. Microbial pesticides consist of a microorganism (e.g., a bacterium, fungus, virus, or protozoan) as the active ingredient. Guidance for conducting the studies listed exist in EPA's Test Guidelines for Pesticides and Toxic Substances^1^ (Leahy et al., 2014).

## European Union (EU) Regulatory Framework for Plant Protection Products

The authorization process of plant protection products is already outlined in Schenkel and Gathmann (2021). The general principles of the European authorization process are again described hereafter to ease the comparison between the U.S. and the EU in this paper.

In the EU, in general, any plant protection product (PPP)—i.e., a pesticide that protects crops or other useful plants—placed on the market needs an authorization. The legal framework is defined in the Regulation (EC) No 1107/2009 (EC, 2009). The authorization process is divided in approval of the active substance and in the authorization of the PPP. A precondition for the authorization of a PPP is an approval of the active substance (a.s.) by the EU Commission based on a risk assessment led by the European Food Safety Authority (EFSA). A PPP containing an approved active substance is assessed and authorized by the Member States (MS). To enhance the efficiency of the authorization process, the EU is divided in three zones, the Northern, Central, and Southern zone (EC, 2009). The risk assessment of a PPP is conducted by one Member State [zonal rapporteur Member State (zRMS)] for the whole zone. In principle, MS of the same zone are forced to take over the results of the assessment and the decision of the zRMS. However, MS can make claims on national ecological or agricultural specificities to decide on dividing risk management options for their country.

Data requirements for assessing active substances are laid down in Regulation (EC) No 283/2013 (EC, 2013a) and for PPPs in Regulation (EC) No 284/2013 (EC, 2013b), respectively. Uniform principles for evaluation and authorization of plant protection products are laid down in the implementing Regulation (EU) No 546/2011 (EC, 2011a). The goal of the regulations is to achieve a high level of protection of human and animal health as well as the environment in all Member States. The regulations are complemented by guidance documents produced by OECD, EPPO, and EFSA to describe the methodological requirements for the risk assessment of active substances and pesticide products.

In principle the Regulation (EC) No 1107/2009 (EC, 2009) differentiates between PPPs based on chemicals and microorganisms, where microorganism means any microbiological entity, including lower fungi and viruses, cellular or non-cellular, capable of replication or of transferring genetic material. Specific data requirements for the assessment of microorganisms are laid down for active substances in Part B of Regulation (EC) No 283/2013 (EC, 2013a) and for PPP in Part B of Regulation (EC) No 284/2013 (EC, 2013b), respectively. Additionally, further categories of PPPs are defined in the Regulation (EC) No 1107/2009 (EC, 2009). PPPs classified as low-risk products have to fulfill strict criteria in particular low toxicological potential (and low persistence in the environment (for details see Article 22 Annex II of EC, 2009). Furthermore, no specific risk mitigation measures should be required following the risk assessment for low-risk PPPs and they have to be sufficiently effective. In practice only a few a.s. are authorized in the EU as low-risk PPPs (see EU, 2021). Another category of PPPs are basic substances. A basic substance is not predominantly used for plant protection purpose but nevertheless it is useful in plant protection either directly or in a product consisting of the substance and a simple diluent. It does not have any inherent capacity to cause endocrine disrupting, neurotoxic or immunotoxic effects and it is not a substance of concern (Article 23, EC, 2009), which means it has no capacity to cause an adverse effect on humans (Article 3, EC, 2009). Currently 23 basic substances are approved in the EU for example beer, *Allium cepa* extraxt or L-cysteine (EU, 2021). A category “biopestides” with divergent data requirements as in the U.S. does not exist in the EU. PPPs based on dsRNA do not fit in any of these categories.

The initial focus of the regulations was set on chemicals as active substances. However, Article 77 of the Regulation (EC) No 1107/2019 (EC, 2009) offers the possibility that “the Commission may … adopt or amend technical and other guidance documents such as explanatory notes or guidance documents on the content of the application concerning microorganisms, pheromones and biological products, for the implementation of this regulation. The Commission may ask the Authority to prepare or to contribute to such guidance documents.” The rationale behind Article 77 might be that data requirements for these product classes deviate from those for chemicals. Such specific adaptions on the data requirement are already implemented for microorganisms, pheromones and botanicals (EC, 2011b, 2014a,b; EC, 2016). However, no specific guidance documents defining the data requirements for the authorization of dsRNA-based PPPs are in sight, but first considerations and recommendations were presented by OECD (2020).

In summary PPPs based on dsRNA currently have to be considered as chemical PPPs.

## Production and Application Methods of dsRNA-Based Pesticides

For successful commercial application of RNAi for crop protection, it is necessary that products are cost-efficient, safe and can be reliably delivered to the site of action in the target organism. There are several ways of production for sequence specific dsRNA. While chemical synthesis as well as *in vitro* transcription is rather costly, dsRNA production in microorganisms is much less expensive (Dalakouras et al., 2020; Taning et al., 2020). However, considerations for microbial production include possible by-products from fermentation and the added issue of genetically modified organisms (GMO). In addition to the usual quality control to ensure dsRNA purity, bacterial production systems require particular care in order to exclude potential contaminants and living GMOs. Recently cell-free platforms for dsRNA synthesis have been established which enable the inexpensive GMO-free production of large dsRNA amounts sufficient for usage in agricultural applications (Taning et al., 2020).

It has been found repeatedly that naked dsRNA is not stable long enough after foliar or soil application to ensure long-lasting plant protection (Mitter et al., 2017; Qiao et al., 2021), especially under field conditions where UV light, rain-fall and microbial degradation account for its rapid degradation and inactivation (Parker et al., 2019; Bachman et al., 2020). Therefore, stabilizing formulations may be necessary for successful usage of dsRNA in topical spray-induced gene silencing (SIGS) applications. Mitter et al. (2017) used positively charged layered double hydroxyde (LDH) clay nanosheets as dsRNA carrier (BioClay) in order to protect dsRNA from degradation after foliar application. Other potential dsRNA carriers that were found to increase the persistence of dsRNA in soil are cationic polymers (Whitfield et al., 2018).

Depending on the target pest or pathogen there are different ways of exogenous application of dsRNA (Dalakouras et al., 2020). Most straightforward is SIGS that was shown to be effective for the control of leaf-feeding insects and against certain phytopathogenic fungi (Koch et al., 2016; San Miguel and Scott, 2016; Wang et al., 2016), when dsRNA uptake into plant cells is not required. If xylem sap-feeding insects or xylem-residing pathogens are the target, more laborious application techniques are required. Dalakouras et al. (2018) demonstrated that double-stranded hairpin (hp) RNA could be delivered into the xylem of woody plants by trunk injection, where it was systemically transported. With conventional SIGS and trunk injection, dsRNA is not taken up by plant cells and thus is not processed into effective small interfering RNAs (siRNAs) within the plant. For the control of plant pathogenic viruses, however, dsRNA or effector siRNAs need to be delivered into plant cells in order to enter the plant RNA silencing machinery. Entry into plant cells is usually not easily achieved due to the cuticule and the plant cell wall, which act as physical barriers for nucleic acids. For successful delivery of RNAi inducers, mechanical wounding of plant tissues is required. This can be achieved by high-pressure spraying, as was shown by Dalakouras et al. (2016) or by special carrier molecules facilitating cellular uptake. Examples for carrier molecules successfully used for plant cell delivery are positively charged peptides (Numata et al., 2014), cationic fluorescent nanoparticles (Jiang et al., 2014), DNA nanostructures (Zhang et al., 2019), and carbon nanotubes (Demirer et al., 2020). These carriers also provide some protection against RNase degradation inside the plant.

Besides carrier molecules or formulations for the enhancement of environmental stability and plant cellular uptake, additional RNA modifications and/or carrier compounds like chitosan, carbon quantum dots (CQD), or silica nanoparticles may be used to enhance cellular intake by insects (Das et al., 2015). Thereby the range of RNAi-based pesticide control may be extended to pest organisms that are less susceptible to RNAi.

## Specific Characteristics of dsRNA-Based Pesticides

There are several factors to consider regarding the data needed to support dsRNA-based pesticides/PPPs due to their mode of action. In this section we highlight some aspects which might alter data needs for the risk assessment and decision making. A detailed overview about the state of the art is given in the OECD Working paper “Considerations for the Environmental Risk Assessment of the Application of Sprayed or Externally Applied ds-RNA-Based Pesticides” (OECD, 2020).

Due to the sequence-specific interaction of siRNAs with complementary target RNA in RNAi (Fire et al., 1998; Baulcombe, 2004), this mode of action can be highly specific for a target organism or a group of target organisms. Yet, potential effects on non-target organisms (NTOs) due to partial sequence homologies of dsRNA and transcription products in NTOs have to be considered (Christiaens et al., 2018). There may be differences between organisms in terms of how the RNAi machinery functions in relation to base pair mismatches and there are scientific uncertainties about the rules determining interactions between siRNA and mRNA. However, current research on RNAi mechanisms, generation of genomic data libraries for relevant species, and design of algorithms to make reliable predictions are expected to increase the usability of bioinformatics data in the prediction of potential effects on NTOs. Under these conditions, bioinformatics models, and programmes that can access a database with a large selection of key sequences can help in identifying off-target sequences to minimize the risk of potential off-target hits. However, bioinformatics can only be one component of the consideration. Risk assessment is also strongly influenced by biological assay data and an overall understanding of the biological and ecological systems in which the dsRNA will be used (Fletcher et al., 2020). Sequence information and bioinformatics can inform the selection and prioritization of non-target species for toxicity and effects testing. Bioinformatics should be augmented by an empirical approach—to introduce dsRNA (that is perfectly complementary to the selected gene in a target organism) to a range of other organisms, starting with close relatives and then moving outwards, to see how more phylogenetically-distant organisms respond (Bachman et al., 2013; Romeis and Widmer, 2020).

Non-Target organism toxicity study protocols for addressing risk with dsRNA-based products require some revisions compared to how they are carried out for biochemical pesticides because dsRNA-based pesticides often take longer to display efficacy. Any evaluation needs to account for this time lag by extending the study observation period. For organisms that have been demonstrated to be responsive to environmental RNA, consideration of life cycle studies (growth, development, and reproduction) and studies on other non-lethal effects should be considered. The importance of sub-lethal endpoints in addition to mortality has been stressed by Romeis and Widmer (2020), who suggest an approach on how to conduct non-target studies for dsRNA-based plant protection products.

In the evaluation of environmental risks from the application of dsRNA-based pesticides, the distribution, fate, stability, and persistence of the dsRNA following product application in the environment will be important (OECD, 2020). The study by Dubelman et al. (2014) shows a rapid degradation of the DvSnf/ dsRNA transcript derived from a genetically modified maize variety in different agricultural soils. Bachman et al. (2020) report a rapid decline in the concentration of foliar applied dsRNA under field conditions with 95% reduction after 3 days. In a recent study, Parker et al. (2019) found that decrease of dsRNA in soil was due to both adsorption to soil and to chemical and microbial degradation. However, the data regarding the environmental fate of dsRNA are still somewhat limited. According to USEPA (2013) the distribution and fate of dsRNA in the environment will depend on the following factors: application rate of active ingredient, application timing, application method, number of applications, off-site movement of applied dsRNA, and the stability and persistence of exogenously applied dsRNA following application.

Some insects, especially of the order Coleoptera (beetles), have been shown to be very sensitive to dsRNA (Baum and Roberts, 2014). Therefore, even small amounts of ingested dsRNA with target sequence homology can induce RNAi which may cause insect mortality. However, there are large differences in the response of insects to ingested dsRNA. Insects of the order Diptera (flies and mosquitoes), Hemiptera (aphids, hoppers, stinkbugs), and Lepidoptera (moths and butterflies), respond to dsRNA with greater variability compared to beetles (Cooper et al., 2019). Additionally, differences in the response can also be found within the same order between species, life stages, genes, and tissues (reviewed in Christiaens et al., 2020). The current knowledge suggests that dsRNA based products might be very specific for an insect genus or species, respectively. The mechanisms triggering the sensitivity of different species to RNAi are not fully understood yet. The potential use of RNAi technology for pest and plant pathogen control is influenced by the cellular uptake of dsRNA (Cooper et al., 2019; Wytinck et al., 2020). Starting with the uptake process and followed by the behavior of the dsRNA in the organism there are many steps that will affect RNAi efficiency. Cooper et al. (2019) collected different hypotheses which were proposed to explain the observed differences in RNAi efficiency among insects. These factors are instability of dsRNA, incomplete dsRNA internalization, deficient core RNAi machinery, impaired systemic spreading of the RNAi signal and refractory target genes.

The problems identified for insects can be similarly transferred to other fields of dsRNA-based PPP applications. While it has been well-known for some time that certain fungi like *Ustilago maydis* and *Saccharomyces cerevisiae* lack core RNAi components (Drinnenberg et al., 2011), recent findings by Qiao et al. (2021) suggest large differences in dsRNA uptake between fungal pathogens. To counter some of the issues, producers are likely to use carriers and formulations containing synergists or co-formulants that stabilize dsRNA in the environment and enhance transport into the cells of target pests. Special attention should be paid to how barriers to uptake are proposed to be overcome for the target organism. Information and/or studies on the impact of carriers and formulations on uptake and environmental persistence are important to characterize exposure to the dsRNA (OECD, 2020).

Due to their relevance for gene regulation and virus defense in plants and animals, dsRNAs as well as their processing products are natural components of food and feed (Dávalos et al., 2019). Therefore, dsRNAs have a long history of safe consumption by humans and other vertebrates (OECD, 2020). Exposition of humans and farm animals after oral uptake of dsRNA or siRNA has been reported to be negligible because of degradation and multiple barriers in the gastrointestinal tract of mammals (O'Neill et al., 2011; Petrick et al., 2013). As is stated in the OECD Working paper (2020), the common occurrence of effective physiological and biochemical barriers among mammals and probably among other vertebrates is likely a consequence of the widespread presence of RNAs in the environment.

## Classification and Authorization Procedure of dsRNA-Based Pesticides for Plant Protection According to U.S. and EU Regulations

While data requirements specific to sprayed or externally applied dsRNA-based pesticides have not been enacted in the U.S., EPA bases its requirements for these pesticides on the biochemical pesticide requirements, outlined in Subpart U—Biochemical Pesticides of 40 CFR Part 158^2^. Often, the technical grade material of the pesticide is tested in mammalian and non-target organism toxicity studies along with product formulations for acute mammalian toxicity and irritation studies. Some of this data may be appropriate to waive according to 40 CFR Part 158.45^3^. Further, data not listed in Subpart U for Biochemical Pesticides that is more specific to sprayed or externally applied dsRNA-based pesticides may be required according to 40 CFR Part 158.75^4^. Where specific product formulations impact barriers to and uptake of the dsRNA, such additional data might include product-specific formulation non-target organism toxicology testing to better characterize the potential for hazard.

If a living genetically engineered organism is used in the manufacturing process to produce a sprayed or externally applied dsRNA-based pesticide, but is not viable in the final product, it would be considered a pesticide intermediate in the US under the Toxic Substances Control Act (TSCA). Pesticide intermediates are subject to oversight under TSCA and would likely require submission of a Microbial Commercial Activity Notice (MCAN) before initiating manufacture (Wozniak et al., 2013).

As outlined above, it can be expected that dsRNA-based PPPs will have different properties than the chemicals mostly used as active substances in PPPs until now. Adaptations of the EU data requirements for the risk assessment might be reasonable for different reasons (see previous chapter). In the EU applications of PPPs with dsRNA as active substance are expected in the coming years. Therefore, the Competent Authorities in the EU should start intensive discussion how the risk of such products will be adequately assessed in the future. One cannot expect that specific guidance documents will be in place when first applications are submitted in the EU. Experiences show that the development and implementation of such guidelines take several years. Until then, the same data requirements are requested as for chemical PPPs. Possible waivers or adjustments of the risk assessment for specific areas of concern might be decided on case-by-case basis.

In the EU additional regulatory requirements might be taken into account depending on the used production system. In the case of production systems based on GM microorganisms an authorization according to Directive (EC) 2001/18 is needed if it cannot be guaranteed that the GM microorganisms are completely inactivated (EC, 2001). It is important to note that GMO-derived products which do not contain a living organism and are not to be used as food or feed, like topically applied PPPs, are not regulated under GMO regulations within the EU (see also Schenkel and Gathmann, 2021).

## Conclusions

The thoughts we highlight in this paper are pertinent for assessing the risk of sprayed or externally applied dsRNA-based pesticides for plant protection. Further discussion of these regulatory and biosafety issues would help better clarify the risk assessment and risk management framework for these new and innovative products. Sprayed or externally applied dsRNA-based pesticides are in the pipeline and the regulatory authorities will likely be having to assess their risk in the near future.

## Author Contributions

The manuscript is based on presentations of AG and MM at an iPlanta webinar on Regulation issues of RNAi based pesticides, chaired by AD-P. All authors contributed to the writing of the manuscript and approved the submitted version.

## Conflict of Interest

The authors declare that the research was conducted in the absence of any commercial or financial relationships that could be construed as a potential conflict of interest.

## Footnotes

^1^https://www.epa.gov/test-guidelines-pesticides-and-toxic-substances/final-test-guidelines-pesticides-and-toxic.

^2^https://www.ecfr.gov/cgi-bin/text-idx?SID=021c1f5a27d7da2ce852a0f903c6709b&mc=true&node=sp40.26.158.u&rgn=div6.

^3^https://www.ecfr.gov/cgi-bin/text-idx?SID=021c1f5a27d7da2ce852a0f903c6709b&mc=true&node=se40.26.158_145&rgn=div8.

^4^https://www.ecfr.gov/cgi-bin/text-idx?SID=021c1f5a27d7da2ce852a0f903c6709b&mc=true&node=se40.26.158_175&rgn=div8.

## References

[B1] Bachman P. Bolognesi R. Moar W. J. Mueller G. M. Paradise M. S. Ramaseshadri P.. (2013). Characterization of the spectrum of insecticidal activity of a double-stranded RNA with targeted activity against Western Corn Rootworm (*Diabrotica virgifera virgifera* LeConte). Transgenic Res. 22, 1207–1222. 10.1007/s11248-013-9716-5 23748931 PMC3835954

[B2] Bachman P. Fischer J. Song Z. Urbanczyk-Wochniak E. Watson G. (2020). Environmental fate and dissipation of applied dsRNA in soil, aquatic systems and plants. Front. Plant Sci. 11:21. 10.3389/fpls.2020.00021 32117368 PMC7016216

[B3] Bachman P. M. Huizinga K. M. Jensen P. D. Mueller G. Tan J. Uffman J. P.. (2016). Ecological risk assessment for DvSnf7 RNA: a plant-incorporated protectant with targeted activity against western corn rootworm. Regul. Toxicol. Pharmacol. 81, 77–88. 10.1016/j.yrtph.2016.08.001 27494948

[B4] Baulcombe D. (2004). RNA silencing in plants. Nature 431, 356–363. 10.1038/nature02874 15372043

[B5] Baum J. A. Roberts J. K. (2014). Progress Towards RNAi-mediated insect pest management. Adv. Insect Physiol. 47, 249–295. 10.1016/B978-0-12-800197-4.00005-1

[B6] Chakraborty S. Newton A. C. (2011). Climate change, plant diseases and food security: an overview. Plant Pathol. 60, 2–14. 10.1111/j.1365-3059.2010.02411.x

[B7] Christiaens O. Dzhambazova T. Kostov K. Arpaia S. Joga M. R. Urru I.. (2018). Literature review of baseline information on RNAi to support the environmental risk assessment of RNAi-based GM plants. EFSA 15:173. 10.2903/sp.efsa.2018.EN-1424

[B8] Christiaens O. Whyard S. Vélez A. M. Smagghe G. (2020). Double-stranded RNA technology to control insect pests: current status and challenges. Front. Plant Sci. 11:451. 10.3389/fpls.2020.00451 32373146 PMC7187958

[B9] Cooper A. M. W. Silver K. Zhang J. Park Y. Zhu K. Y. (2019). Molecular mechanisms influencing efficiency of RNA interference in insects. Pest Manag. Sci. 75, 18–28. 10.1002/ps.5126 29931761

[B10] Dalakouras A. Jarausch W. Buchholz G. Bassler A. Braun M. Manthey T.. (2018). Delivery of hairpin RNAs and small RNAs into woody and herbaceous plants by trunk injection and petiole absorption. Front. Plant Sci. 9:1253. 10.3389/fpls.2018.01253 30210521 PMC6120046

[B11] Dalakouras A. Wassenegger M. Dadami E. Ganopoulos I. Pappas M. L. Papadopoulou K. (2020). Genetically modified organism-free RNA interference: exogenous application of RNA molecules in plants. Plant Physiol. 182, 38–50. 10.1104/pp.19.00570 31285292 PMC6945881

[B12] Dalakouras A. Wassenegger M. McMillan J. N. Cardoza V. Maegele I. Dadami E.. (2016). Induction of silencing in plants by high-pressure spraying of in vitro-synthesized small RNAs. Front. Plant Sci. 7:1327. 10.3389/fpls.2016.01327 27625678 PMC5003833

[B13] Das S. Debnath N. Cui Y. Unrine J. Palli S. R. (2015). Chitosan, carbon quantum dot, and silica nanoparticle mediated dsRNA delivery for gene silencing in Aedes aegypti: a comparative analysis. ACS Appl. Mater. Interfaces 7, 19530–19535. 10.1021/acsami.5b05232 26291176

[B14] Dávalos A. Henriques R. Latasa M. J. Laparra M. Coca M. (2019). Literature review of baseline information on non-coding RNA (ncRNA) to support the risk assessment of ncRNA-based genetically modified plants for food and feed. EFSA 16:220. 10.2903/sp.efsa.2019.EN-1688

[B15] Demirer G. S. Zhang H. Goh N. S. Pinals R. L. Chang R. Landry M. P. (2020). Carbon nanocarriers deliver siRNA into intact plant cells for efficient gene knockdown. Sci. Adv. 6:eaaz0495. 10.1126/sciadv.aaz0495 32637592 PMC7314522

[B16] Drinnenberg I. A. Fink G. R. Bartel D. P. (2011). Compatibility with killer explains the rise of RNAi-deficient fungi. Science 333:1592. 10.1126/science.1209575 21921191 PMC3790311

[B17] Dubelman S. Fischer J. Zapata F. Huizinga K. Jiang C. Uffman J.. (2014). Environmental fate of double-stranded RNA in agricultural soils. PLoS ONE 9:e93155. 10.1371/journal.pone.0093155 24676387 PMC3968063

[B18] EC (2001). Directive 2001/18/EC of the European Parliament and of the Council of 12 March 2001 on the deliberate release into the environment of genetically modified organisms and repealing Council Directive 90/220/EEC - Commission Declaration. OJ L 106, 1–39.

[B19] EC (2009). Regulation (EC) no 1107/2009 of the European parliament and of the council of 21 October 2009 concerning the placing of plant protection products on the market and repealing Council Directives 79/117/EEC and 91/414/EEC. OJ L 309, 1–50.

[B20] EC (2011a). Commission Regulation (EU) No 546/2011 of 10 June 2011 implementing Regulation (EC) No 1107/2009 of the European Parliament and of the Council as regards uniform principles for evaluation and authorisation of plant protection products. OJ L 155, 127–175.

[B21] EC (2011b). Guidance Document on the Assessment of New Substances Falling Into the Group of Straight Chain Lepidopteran Pheromones (SCLPs) Included in Annex I of Council Directive 91/414/EEC (SANCO/5272/2009 rev. 3 28 October 2011).

[B22] EC (2013a). Commission Regulation (EU) No 283/2013 of 1 March 2013 setting out the data requirements for active substances, in accordance with Regulation (EC) No 1107/2009 of the European Parliament and of the Council concerning the placing of plant protection products on the market. OJ L 93, 1–84.

[B23] EC (2013b). Commission Regulation (EU) No 284/2013 of 1 March 2013 setting out the data requirements for plant protection products, in accordance with Regulation (EC) No 1107/2009 of the European Parliament and of the Council concerning the placing of plant protection products on the market. OJ L 93, 85–152.

[B24] EC (2014a). Guidance Document for the Assessment of the Equivalence of Technical Grade Active Ingredients for Identical Microbial Strains or Isolates Approved Under Regulation (EC) no 1107/2009 (SANCO/12823/2012 –rev. 4,12 December 2014).

[B25] EC (2014b). Guidance Document on Botanical Active Substances Used in Plant Protection Products (SANCO/11470/2012– rev. 8 20 March 2014).

[B26] EC (2016). Guidance Document on Semiochemical Active Substances and Plant Protection Products (SANTE/12815/2014 rev. 5.2 May 2016).

[B27] EU (2021). EU Pesticide Data Base. Available online at: https://ec.europa.eu/food/plant/pesticides/eu/pesticides/database/active/substances/?event=search.as (accessed May 11, 2021).

[B28] Fire A. Xu S. Montgomery M. K. Kostas S. A. Driver S. E. Mello C. C. (1998). Potent and specific genetic interference by double-stranded RNA in *Caenorhabditis elegans*. Nature 391, 806–811. 10.1038/35888 9486653

[B29] Fletcher S. J. Reeves P. T. Hoang B. T. Mitter N. (2020). A perspective on RNAi-Based biopesticides. Front. Plant Sci. 11:51. 10.3389/fpls.2020.00051 32117388 PMC7028687

[B30] Gómez-Barbero M. Berbel J. Rodriguez-Cerezo E. (2008). Bt corn in Spain – the performance of the EU's first GM crop. Nat. Biotechnol. 26, 384–386. 10.1038/nbt0408-384 18392015

[B31] Head G. P. Carroll M. W. Evans S. P. Rule D. M. Willse A. R. Clark T. L.. (2017). Evaluation of SmartStax and SmartStax PRO maize against western corn rootworm and northern corn rootworm: efficacy and resistance management. Pest Manag. Sci. 73, 1883–1899. 10.1002/ps.4554 28195683

[B32] ISAAA (2019). Global Status of Commercialized Biotech/GM crops in 2019: Biotech crops Drive Socio-Economic Development and Sustainable Environment in the New Frontier. Ithaca, NY: ISAAA.

[B33] Jalaluddin N. S. M. Othman R. Y. Harikrishna J. A. (2019). Global trends in research and commercialization of exogenous and endogenous RNAi technologies for crops. Crit. Rev. Biotechnol. 39, 67–78. 10.1080/07388551.2018.1496064 30198341

[B34] Jiang L. Ding L. He B. Shen J. Xu Z. Yin M.. (2014). Systemic gene silencing in plants triggered by fluorescent nanoparticle-delivered double-stranded RNA. Nanoscale 6, 9965–9969. 10.1039/C4NR03481C 25068243

[B35] Koch A. Biedenkopf D. Furch A. Weber L. Rossbach O. Abdellatef E.. (2016). An RNAi-based control of Fusarium graminearum infections through spraying of long dsRNAs involves a plant passage and is controlled by the fungal silencing machinery. PLoS Pathogens 12:e1005901. 10.1371/journal.ppat.1005901 27737019 PMC5063301

[B36] Leahy J. Mendelsohn M. Kough J. Jones R. Berckes N. (2014). Biopesticide oversight and registration at the U.S. Environmental Protection Agency in Biopesticides: state of the art and future opportunities, in ACS Symposium Series, eds Coats. (Washington, DC: American Chemical Society). 10.1021/bk-2014-1172.ch001

[B37] Mendelsohn M. Kough J. Vaituzis Z. Matthews K. (2003). Are Bt crops safe? Nat. Biotechnol. 21, 1003–1009. 10.1038/nbt0903-1003 12949561

[B38] Mitter N. Worrall E. A. Robinson K. E. Li P. Jain R. G. Taochy C.. (2017). Clay nanosheets for topical delivery of RNAi for sustained protection against plant viruses. Nat. Plants 3:16207. 10.1038/nplants.2016.207 28067898

[B39] Numata K. Ohtani M. Yoshizumi T. Demura T. Kodama Y. (2014). Local gene silencing in plants via synthetic dsRNA and carrier peptide. Plant Biotechnol. J. 12, 1027–1034. 10.1111/pbi.12208 24905384

[B40] OECD (2020). Considerations for the Environmental Risk Assessment of the Application of Sprayed or Externally Applied dsRNA-based Pesticides. Series on Pesticides No. 104, ENV/JM/MONO (2020) 26 (Paris).

[B41] O'Neill M. J. Bourre L. Melgar S. O'Driscoll C. M. (2011). Intestinal delivery of non-viral gene therapeutics: physiological barriers and preclinical models. Drug Discovery Today 16, 203–218. 10.1016/j.drudis.2011.01.003 21262379

[B42] Parker K. M. Barragán Borrero V. Van Leeuwen D. M. Lever M. A. Mateescu B. Sander M. (2019). Environmental fate of RNA interference pesticides: adsorption and degradation of double-stranded RNA molecules in agricultural soils. Environ. Sci. Technol. 53, 3027–3026. 10.1021/acs.est.8b05576 30681839

[B43] Perlak F. J. Deaton R. W. Armstrong T. A. Fuchs R. L. Sims S. R. Greenplate J. T.. (1990). Insect resistant cotton plants. Bio/Technology 8, 939–949. 10.1038/nbt1090-939 1366777

[B44] Petrick J. S. Brower-Toland B. Jackson A. L. Kier L. D. (2013). Safety assessment of food and feed from biotechnology-derived crops employing RNA-mediated gene regulation to achieve desired traits: a scientific review. Reg. Toxicol. Pharm. 66, 157–176. 10.1016/j.yrtph.2013.03.008 23557984

[B45] Qiao L. Lan C. Capriotti L. Ah-Fong A. Sanchez J. N. Hamby R.. (2021). Spray-induced gene silencing for disease control is dependent on the efficiency of pathogen RNA uptake. bioRxiv [Preprint]. 10.1101/2021.02.01.429265 PMC8428832 33774895

[B46] Romeis J. Widmer F. (2020). Assessing the risk of topically applied dsRNA-based products to non-target arthropods. Front. Plant Sci. 11:679. 10.3389/fpls.2020.00679 32582240 PMC7289159

[B47] San Miguel K. Scott J. G. (2016). The next generation of insecticides: dsRNA is stable as a foliar-applied insecticide. Pest Manag. Sci. 72, 801–809. 10.1002/ps.4056 26097110

[B48] Schenkel W. Gathmann A. (2021). Regulatory aspects of RNAi in plant production, in RNAi for Improved Crop Performance Plant Improvement and Protection, eds. Mezzetti B. Sweet J. B. Burgos L. (Oxfordshire: CABI), 154–168. 10.1079/9781789248890.0154

[B49] Taning C. N. T. Arpaia S. Christiaens O. Dietz-Pfeilstetter A. Jones H. Mezzetti B.. (2020). RNA-based biocontrol compounds: current status and perspectives to reach the market. Pest Manag. Sci. 76, 841–845. 10.1002/ps.5686 31743573

[B50] Tenllado F. Diaz-Ruiz J. R. (2001). Double-stranded RNA-mediated interference with plant virus infection. J. Virol. 75, 12288–12297. 10.1128/JVI.75.24.12288-12297.2001 11711619 PMC116125

[B51] USEPA (2013). White Paper on RNAi Technology as a Pesticide: Problem Formulation for Human Health and Ecological Risk Assessment. (Submitted to the FIFRA Science Advisory Panel for review on comment. October 29, 2013). Available online at: https://www.regulations.gov/document/EPA-HQ-OPP-2013-0485-0011 (accessed May 11, 2021).

[B52] Wang M. Weiberg A. Lin F. M. Thomma B. P. Huang H. D. Jin H. (2016). Bidirectional cross-kingdom RNAi and fungal uptake of external RNAs confer plant protection. Nat. Plants 2:16151. 10.1038/nplants.2016.151 27643635 PMC5040644

[B53] Whitfield R. Anastasaki A. Truong N. P. Cook A. B. Omedes-Pujol M. Loczwenski Rose V.. (2018). Efficient binding, protection, and self-relaese of dsRNA in soil by linear and star cationic polymers. ACS Macro Lett. 7, 909–915. 10.1021/acsmacrolett.8b00420 35650964

[B54] Worrall E. A. Bravo-Cazar A. Nilon A. T. Fletcher S. J. Robinson K. E. Carr J. P.. (2019). Exogenous applicaton of RNAi-inducing double-stranded RNA inhibits aphid-mediated transmission of a plant virus. Front. Plant Sci. 10:265. 10.3389/fpls.2019.00265 30930914 PMC6429036

[B55] Wozniak C. McClung G. Gagliardi J. Segal M. Matthews K. (2013). Regulation of genetically engineered microorganisms under FIFRA, FFDCA and TSCA, in Regulation of Agricultural Biotechnology: The United States and Canada, eds Wozniak C. A. McHughen A. (Dordrecht: Springer), 57–94. 10.1007/978-94-007-2156-2_4

[B56] Wytinck N. Manchur C. L. Li V. H. Whyard S. Belmonte M. F. (2020). dsRNA uptake in plant pests and pathogens: insights into RNAi-based insect and fungal control technology. Plants 9:1780. 10.3390/plants9121780 33339102 PMC7765514

[B57] Zhang H. Demirer G. S. Zhang H. Ye T. Goh N. S. Aditham A. J.. (2019). DNA nanostructures coordinate gene silencing in mature plants. Proc. Natl. Acad. Sci U.S.A. 116, 7543–7548. 10.1073/pnas.1818290116 30910954 PMC6462094

